# Downregulation of *PTPRT* elevates the expression of survivin and promotes the proliferation, migration, and invasion of lung adenocarcinoma

**DOI:** 10.1186/s12885-024-11840-7

**Published:** 2024-01-12

**Authors:** Chao Chen, Haozhen Liu, Yanling Li, Qumiao Xu, Jixian Liu

**Affiliations:** 1 Department of Thoracic Surgery, Peking University Shenzhen Hospital, Shenzhen Peking University, The Hong Kong University of Science and Technology Medical Center, Shenzhen, 518035 China; 2 BGI Research, Hangzhou, 310030 China

**Keywords:** *PTPRT*, Cell cycle, *BIRC5*, Lung adenocarcinoma

## Abstract

**Background:**

Receptor-type tyrosine-protein phosphatase T (PTPRT) is a transmembrane protein that is involved in cell adhesion. We previously found that *PTPRT* was downregulated in multiple cancer types and the mutation of *PTPRT* was associated with cancer early metastasis. However, the impacts of *PTPRT* downregulation on tumour proliferation, invasion, and clinical interventions such as immune checkpoint inhibitor (ICI) therapies remained largely unknown.

**Methods:**

Gene expression data of non-small cell lung cancer (NSCLC) samples from The Cancer Genome Atlas database were downloaded and used to detect the differential expressed genes between PTPRT*-*high and PTPRT*-*low subgroups. Knockdown and overexpress of *PTPRT* in lung cancer cell lines were performed to explore the function of *PTPRT *in vitro. Western blot and qRT-PCR were used to evaluate the expression of cell cycle-related genes. CCK-8 assays, wound-healing migration assay, transwell assay, and colony formation assay were performed to determine the functional impacts of *PTPRT* on cell proliferation, migration, and invasion. KM-plotter was used to explore the significance of selected genes on patient prognosis.

**Results:**

*PTPRT* was found to be downregulated in tumours and lung cancer cell lines compared to normal samples. Cell cycle-related genes (*BIRC5*, *OIP5*, and *CDCA3*, etc.) were specifically upregulated in PTPRT-low lung adenocarcinoma (LUAD). Modulation of *PTPRT* expression in LUAD cell lines affected the expression of *BIRC5* (survivin) significantly, as well as the proliferation, migration, and invasion of tumour cells. In addition, low *PTPRT* expression level was correlated with worse prognosis of lung cancer and several other cancer types. Furthermore, *PTPRT* downregulation was associated with elevated tumour mutation burden and tumour neoantigen burden in lung cancer, indicating the potential influence on tumour immunogenicity.

**Conclusion:**

Our findings uncovered the essential roles of *PTPRT* in the regulation of proliferation, migration, and invasion of LUAD, and highlighted the clinical significance of *PTPRT* downregulation in lung cancer.

**Supplementary Information:**

The online version contains supplementary material available at 10.1186/s12885-024-11840-7.

## Background

Receptor-type tyrosine-protein phosphatase T (PTPRT) is a transmembrane protein encoded by the *PTPRT* gene [1]. Previous studies have shown that *PTPRT* was involved in cell adhesion and phosphorylation of signal transducer and activator of transcription (STAT) [2, 3]. Furthermore, as a well-known tumour suppressor gene, mutations in *PTPRT* were associated with the occurrence of cancer [4]. *PTPRT* was found to be frequently mutated in multiple cancer types, especially in cutaneous melanoma, gastric adenocarcinoma, endometrial cancer, and lung cancer [5, 6]. Through the analysis of tumour genomic and clinical data, our previous study also supported that mutation events in *PTPRT* might serve as candidate markers of tumour early metastasis [7].

In recent years, immune checkpoint inhibitors (ICIs) play important roles in the treatment of cancers [8–11], while tumour mutation burden (TMB) was considered to be a prominent marker for responses to ICIs therapy [12–15]. Previous studies have shown that *PTPRT* mutations were associated with higher TMB and can predict the efficacy of ICIs [16, 17]. They also found that the mutation of *PTPRT* was associated with enhanced infiltration of CD8^+^ T cells and M1-macrophages [17]. These studies suggested that the dysfunction of *PTPRT* may be closely related to tumour immunogenicity. Our previous study have found that *PTPRT* was downregulated in a variety of cancers [7], but the effects and mechanisms of *PTPRT* downregulation on proliferation, migration, invasion, and tumour immunogenicity of lung cancer are still poorly understood.

In this study, through analysis of the gene expression data of TCGA lung adenocarcinoma (LUAD) and lung squamous cell carcinoma (LUSC) from The Cancer Genome Atlas (TCGA) database, we depicted the genes and pathways associated with *PTPRT* downregulation*.* We then validated the functional impacts of *PTPRT* expression on cell proliferation, migration and invasion through in vitro experiments using lung cancer cell lines. We also highlighted the role of *PTPRT* in modulating the expression of cell cycle-related genes, especially for *BIRC5*. Finally, the potential value of *PTPRT* as a biomarker for the prognosis of lung cancer was explored.

## Materials and methods

### Samples and data collection

The expression data of TCGA pan-cancer samples were obtained from the UCSC Xena database (https://toil-xena-hub.s3.us-east-1.amazonaws.com/download/tcga_RSEM_gene_tpm.gz). The clinical information of TCGA samples ("TCGA-CDR-Supplemental Table S1.xlsx”), containing the tumour types (such as LUAD and LUSC) was downloaded from the PanCanAtlas database (https://gdc.cancer.gov/about-data/publications/pancanatlas). Then transcripts per million (TPM) values of 509 LUAD and 479 LUSC samples were extracted. Four LUAD cell lines (A549, H1299, H1975, H838) and human normal lung epithelial cells BEAS-2B were purchased from the American Type Culture Collection (ATCC) for in vitro experiments.

### Detection of differentially expressed genes (DEGs)

The DEGs between the PTPRT-high and PTPRT-low subgroups were detected by the limma package (v3.52.3). We divided the samples into PTPRT-high and PTPRT-low subgroups using upper and lower quartile TPM values of *PTPRT* in the TCGA-LUAD (upper quartile: 0.37; lower quartile: 0.02) or TCGA-LUSC (upper quartile: 0.94; lower quartile: 0.04) cohort. The parameter “decideTestsDGE(adjust.method = "fdr", *p*.value = 0.01,lfc = 1)” was used to detect DEGs and ggplot2 (version 3.3.6) was used for visualization.

### Functional enrichment analysis

The DEGs (FDR < 0.01 & |log2(foldchange)|> 1) between the PTPRT-high and PTPRT-low subgroups were selected for functional enrichment analysis. The Gene Ontology (GO) and KEGG pathway enrichment analysis were performed using the DAVID database (https://david.ncifcrf.gov/tools.jsp) [18]. Gene symbols of DEGs were used as input, and functional terms with “FDR < 0.05” were defined as significant enrichment.

### Quantitative reverse transcription polymerase chain reaction (qRT-PCR)

Total RNA was extracted using the RNA extraction kit (#RNAfast200, Fastagen, China), and the reverse transcription and qPCR were conducted using ReverTra Ace qPCR RT Kit and SYBR Green Realtime PCR Master Mix (#FSQ-101, #QPK-212, Toyobo Life Science, China) according to the manufacturer's instructions. The relative gene expression was examined by the comparative Ct method using 2^−△△Ct^. Primers were listed in supplementary Table S1.

### Western blot

Cell samples were lysed with cold RIPA lytic buffer (#P0013B, Beyotime, China), and the extracted proteins were quantified using Pierce BCA Protein Assay Kit (#P0009, Beyotime, China). After electrophoresis was performed on a sodium dodecyl sulphate polyacrylamide gel (SDS-PAGE), the blots were transferred to a polyvinylidene difluoride membrane (#IPVH00010, Millipore, America). The membranes were incubated with diluted primary antibodies (survivin, #10,508–1-AP, 1:1000, Proteintech; PTPRT, #PA5-18,304, 1:500, Invitrogen) and GAPDH (#60,004–1-Ig, 1:16,000, Proteintech) at 4 °C overnight, and then the second antibody was added and incubated at room temperature for 30 min. ECL reagent (#RM00020, ABclonal, China) was added to the membranes to visualize the immunoreactive protein bands. Notably, the marker (Shanghai Yase Biomedical Technology Co., LTD., WJ102) was not able to be developed. Therefore, the position of the marker on the Kodak film after exposure was determined by comparing the color bands on the acetate fiber film using SDS-PAGE horizontal electrophoresis (Supplementary Fig. 1– 4).

### RNA interference

To knock down *PTPRT* expression, different siRNAs (si-PTPRT-92, si-PTPRT-305, si-PTPRT-921) were designed and synthesized (GenePharma). A scrambled siRNA was used as the negative control (si-NC). The sequences of siRNAs were listed in Table S2. Cells were transiently transfected with 100 pmol of siRNAs using Lipofectamine 3000 (Invitrogen). After transfection for 48 h, the cells were collected for further experiments.

### Overexpression of *PTPRT*

The coding sequence of *PTRRT* was synthesized and inserted into pcDNA™ 3.1Zeo ( +) plasmid for overexpression of *PTPRT* (OE-PTPRT). Empty pcDNA™ 3.1Zeo ( +) plasmid-transfected cells as a control (OE-NC). Transfected cells were collected 24h later, and total RNA was extracted, followed by detection of *PTRRT* expression by qRT-PCR.

### CCK-8 and colony formation assay

The treated cells were digested and counted, and 1000 cells were inoculated into 96-well plates per well, cultured in a 37℃, 5% CO2 incubator for 0, 48h, and 72h. CCK-8 solution (#CA1210, Solarbio Life Science, China) was added to each well and was further incubated for 1-2h. Then the absorbance at 450nm was measured with a microplate reader (#ST-360, Kehua Bio-engineering Co., China) to compare the cell proliferation rates. For the colony formation assay, 400–1000 cells were seeded into 6-well plates and cultured for 10–14 days. Cells were then fixed with 4% paraformaldehyde (#30,188,928, Sinopharm, China) and stained with crystal violet staining buffer (#C0121-100ml, Beyotime, China) to allow the counting of cell colonies.

### Wound-healing migration assay

About 5 × 10^5^ cells were cultured in 6-well plates, and after monolayers of cells were formed, they were then gently scratched with a sterilized pipette tip. Then the cells were washed three times with PBS to remove the scratched cells and continued in culture with fresh medium added at 37℃ and 5% CO^2^ for 24 h. Photographs were taken in three random microscopic fields at 0 h, 24 h, 48h, and 72h.

### Transwell assay

Transwell assays were conducted as previously described with modification [19]. Briefly, 1 × 10^4^ cells were resuspended in a 10% FBS medium and inoculated into the upper wells of transwells. After incubation at 37℃ and 5% CO^2^ for 24 h, invasive cells to the lower wells were fixed with 4% paraformaldehyde and stained with 1% crystal violet (#DL22040, DLM, China) for 20 min. The number of invasive cells was counted in three microscopic fields.

### Survival analysis

The survival analysis between PTPRT-high and PTPRT-low subgroups was performed on the “mRNA RNA-seq: Pancancer” module of the KM-plotter (http://kmplot.com) [20], “Auto select best cutoff” and “Follow up threshold: 60 months” were used to calculate the prognostic curves for these two subgroups.

### The correlation between *PTPRT* and tumour immunogenicity

The mutation data of the TCGA pan-cancer cohort was downloaded from the PanCanAtlas database (https://gdc.cancer.gov/about-data/publications/pancanatlas). TMB was defined as the number of non-silent mutations (missense, nonsense, insertion/deletion, splice-site) per sample. The LUAD and LUSC samples were extracted according to tumour types. The neoantigen data of LUAD and LUSC samples was acquired from the Cancer Immunome Atlas (TCIA) database (https://www.tcia.at/home). TNB was defined as the number of neoantigens per sample.

The comparison of TMB between PTPRT-high and PTPRT-low subgroups (Fig. 7A, B, E, F) was conducted using the Comprehensive Analysis on Multi-Omics of Immunotherapy in Pan-cancer (CAMOIP) database (https://www.camoip.net/). The correlation of *PTPRT* expression and TMB/TNB was calculated with the “Spearman” method, using samples with both TMB/TNB and TPM values (Fig. 7C, D, G, H).

### Statistical analysis

R software (v4.2.1, http://www.r-project.org) was used for all statistical analyses. The correlation between *PTPRT* and other genes was calculated with the “cor.test” function, and the “Spearman” method was used to calculate the correlation coefficient. *P* < 0.05 was considered statistically significant if not otherwise specified.

## Results

### Differential expressed genes (DEGs) and pathways associated with *PTPRT*

**Fig. 1:**
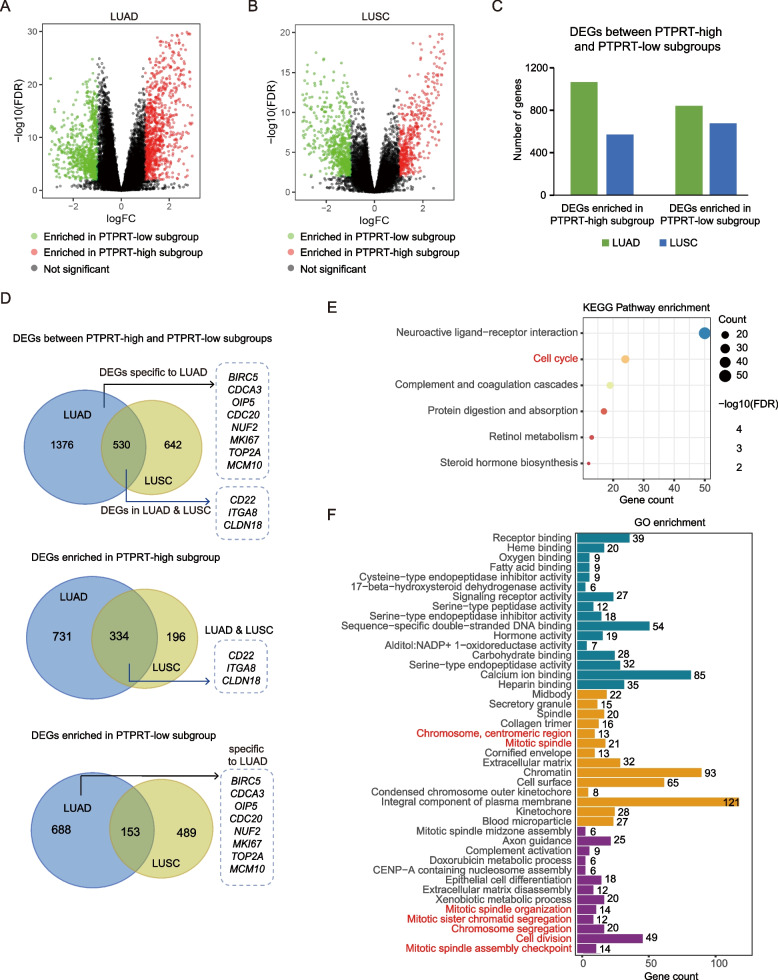
The differential expressed genes (DEGs) between PTPRT-high and PTPRT-low subgroups in TCGA-LUAD and TCGA-LUSC cohorts and functional enrichment analysis. **A **DEGs between PTPRT-high and PTPRT-low subgroups in TCGA-LUAD cohort. **B **DEGs between PTPRT-high and PTPRT-low subgroups in TCGA-LUSC cohort. **C **DEGs number in TCGA-LUAD and TCGA-LUSC cohorts. **D **Comparison of DEGs between TCGA-LUAD and TCGA-LUSC cohorts. Top, Venn diagram showing DEGs between TCGA-LUAD and TCGA-LUSC cohorts. Middle, Venn diagram showing DEGs enriched in PTPRT-high subgroup between TCGA-LUAD and TCGA-LUSC cohorts. Bottom, Venn diagram showing DEGs enriched in PTPRT-low subgroup between TCGA-LUAD and TCGA-LUSC cohorts. **E **KEGG pathway enrichment of DEGs specific to LUAD. **F **Gene ontology enrichment of DEGs specific to LUAD. The terms marked in red in **E** and **F** are cell cycle or cell division-related pathways/biological processes

To reveal genes and pathways associated with the downregulation of *PTPRT*, we downloaded the gene expression data of LUAD (*N* = 509) and LUSC (*N* = 479) samples from TCGA database, which were divided into PTPRT-high and PTPRT-low subgroups according to the upper and lower quartile TPM values of *PTPRT*, respectively. DEGs between PTPRT-high and PTPRT-low subgroups were identified with the limma package (v3.52.3) (Fig. 1A-C, Table S3- 4). More DEGs were found in the TCGA-LUAD group, with 1376 DEGs unique to the TCGA-LUAD group alone (Fig. 1D). Interestingly, some cell adhesion-associated DEGs (such as *ITGA8*, *CD22*, and *CLDN18*) were found in both TCGA-LUAD and TCGA-LUSC cohorts and enriched in PTPRT-high subgroup (Fig. 1D), which was consistent with the previously reported function of *PTPRT* in cell adhesion [21]. On the other hand, many cell cycle-related DEGs (such as *BIRC5*, *OIP5*, *NUF2*, *MCM10, MKI67, and TOP2A*) were detected only in the TCGA-LUAD cohort and enriched in the PTPRT-low subgroup (Fig. 1D). To further explore the function of these DEGs, we conducted GO and KEGG pathway enrichment analysis [22]. Not surprisingly, DEGs specific to LUAD were enriched in cell cycle pathway or cell cycle-related terms, such as cell division, chromosome segregation, mitotic spindle organization, mitotic spindle, and mitotic spindle assembly checkpoint (Fig. 1E, F, Table S5- 6). Given that there are more DEGs associated with *PTPRT* in LUAD than in LUSC, and these DEGs are enriched in cell cycle-related pathways, we hypothesized that the downregulation of *PTPRT* in LUAD may have an impact on the cell proliferation of cancer cells. Therefore, we next focused on the influence of downregulation of *PTPRT* on the progression of LUAD.

**Fig. 2:**
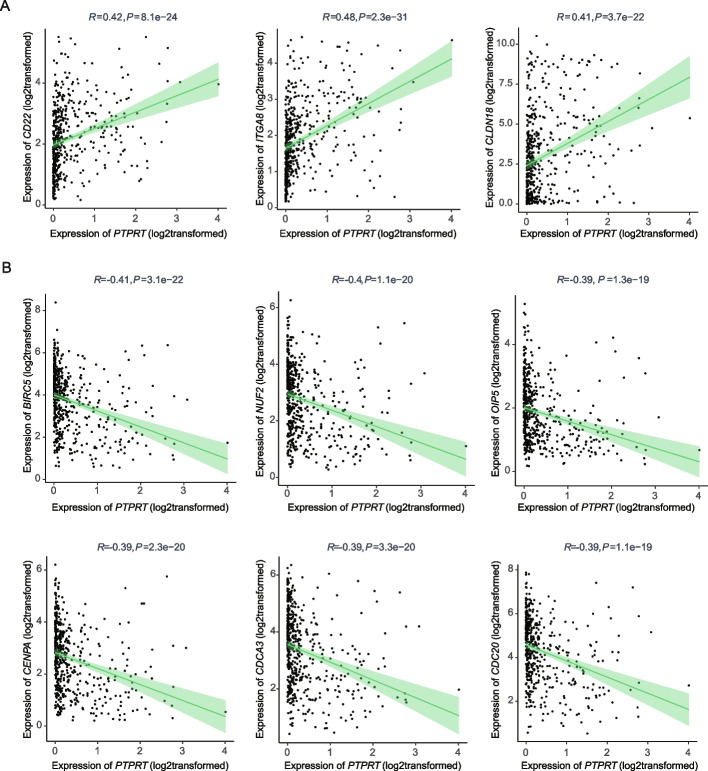
Genes significantly correlated with *PTPRT* in TCGA-LUAD. **A** The cell adhesion-related genes were positively correlated with *PTPRT*. **B **The cell cycle-related genes were negatively correlated with *PTPRT*. Spearman correlation was determined

We further explored the expression correlation between *PTPRT* and genes involved in the cell adhesion or cell cycle pathways. As expected, genes in the cell adhesion pathway (such as *CD22, ITGA8, CLDN18*) were significantly positively correlated with *PTPRT* (all with R > 0.4, *P* < 0.01; Fig. 2A) Specifically, DEGs upregulated in the PTPRT-low subgroup specific to the TCGA-LUAD cohort were negatively correlated with *PTPRT* (Fig. 2B) in TCGA-LUAD, but not in TCGA-LUSC, including *BIRC5* (R = -0.41, *P* < 0.001), *NUF2* (R = -0.4, *P* < 0.001), *OIP5* (R = -0.39, *P* < -0.001), *CENPA* (R = -0.39, *P* < 0.001), *CDC20* (R = -0.39, *P* < 0.001), and *CDCA3* (R = -0.39, *P* < 0.001), which were genes associated with the cell cycle pathway. The essential antiapoptotic gene *BIRC5 (*also known as survivin*)* was abundantly expressed in adenocarcinoma and involved in promoting cell proliferation and preventing cell apoptosis [23]. *NUF2*, *OIP5* and *CDC20* were key players in the regulation of cell division [24–26]. Together, these results showed that *PTPRT* expression was significantly correlated with multiple genes in the cell adhesion and cell cycle pathways in lung cancer, especially in LUAD.

### *PTPRT* inhibited the cell proliferation and expression of BIRC5 in vitro

**Fig. 3:**
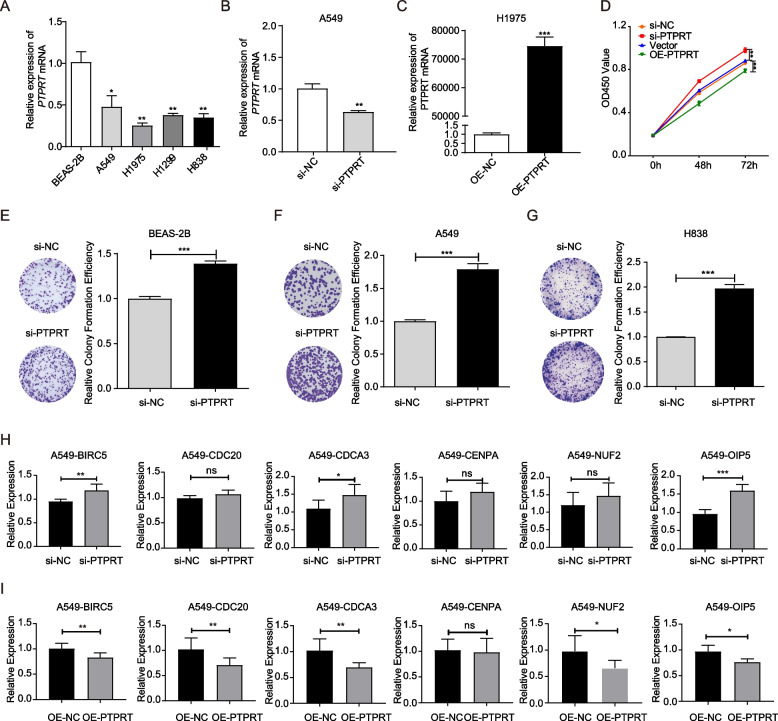
In vitro validation of the influence of *PTPRT* with cell proliferation in LUAD cell lines. **A** qRT-PCR of *PTPRT* in four LUAD cell lines and a human bronchial epithelial cell lines BEAS-2B. **B** Knockdown of *PTPRT* (si-PTPRT-921) in A549 cells. **C** Overexpress of *PTPRT* (OE-PTPRT) in H1975 cells compared with negative control (OE-NC). **D** Cell proliferation curves after 4 different treatments (si-NC, si-PTPRT, OE-NC, and OE-PTPRT). Data were shown as the mean ± SD (*n* = 3). * *P* < 0.05, ** *P* < 0.01, *** *P* < 0.001 by unpaired Student’s t test. **E**–**G **The changes of colony formation efficiency upon *PTPRT* knockdown in non-malignant lung epithelial cells (E, BEAS-2B) and lung cancer cells (F, A549; G, H838). **H**-**I **The changes of cell cycle-related genes upon *PTPRT* knockdown (**H**) and overexpression (**I**)

We then performed cell-based assays to explore the roles of *PTPRT* on cell proliferation, using four LUAD cell lines (A549, H1975, H1299, and H838). We first confirmed that the expression of *PTPRT* in those LUAD cells was significantly lower compared to the normal BEAS-2B bronchial epithelial cells (Fig. 3A). Among the four tumour cell lines, the expression of *PTPRT* was the highest in A549 and the lowest in H1975. Thus, knockdown or overexpression of *PTPRT* was conducted in A549 or H1975 cells (Fig. 3B, C). As CCK-8 assays showed, *PTPRT*-knocked-down A549 cells displayed enhanced cell proliferation, while *PTPRT*-overexpressing H1975 cells had decreased cell proliferation (Fig. 3D), suggesting an inhibitory role of *PTPRT* in cancer cell proliferating. Furthermore, cell colony formation was increased after the knockdown of *PTPRT* in non-malignant lung epithelial cells (Fig. 3E) or multiple LUAD cells (Fig. 3F, G). These data supported that *PTPRT* functioned as a negative regulator of cell proliferation.

To evaluate whether the cell cycle-related genes were regulated by *PTPRT,* we examined the expression of various genes (*BIRC5*, *OIP5*, *CDC20*, *CDCA3*, *CENPA*, and *NUF2*) by qRT-PCR after *PTPRT* knockdown or overexpression in A549 cells. As expected, the expression of *BIRC5*, *CDCA3*, and *OIP5* was significantly upregulated upon *PTPRT* knockdown (Fig. 3H), while overexpression of *PTPRT* resulted in the downregulation of *BIRC5*, *CDC20*, *CDCA3*, *NUF2*, and *OIP5* (Fig. 3I). These results further suggested the role of *PTPRT* in regulating the cell cycle pathway.

**Fig. 4:**
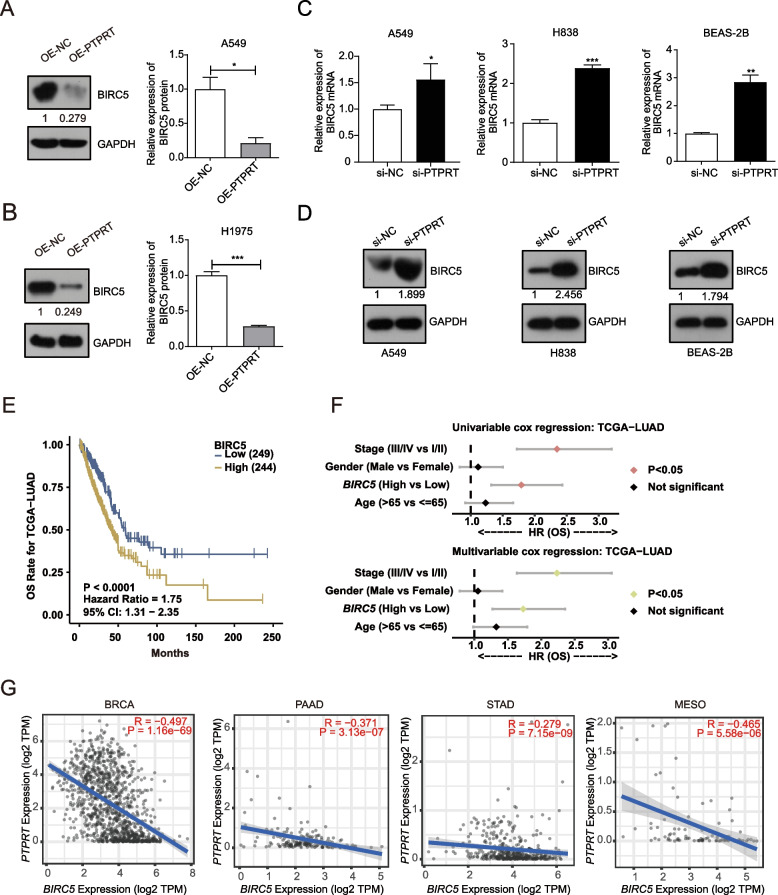
Regulation of *PTPRT* on *BIRC5* and its effect on tumour prognosis. **A**, **B** Overexpression of *PTPRT* led to decreased expression of BIRC5 (survivin) at protein level (*P* = 0.029, two-sided Student's t-test) in A549 (**A**) and H1975 (**B**) cells. Three replicates of Western blots were performed, full-length gels and blots were shown in Supplementary Fig. 1– 2. **C** Increased *BIRC5* mRNA expression in si-PTPRT compared to si-NC cells. **D** Increased *BIRC5* protein level in si-PTPRT compared to si-NC cells, full-length gels and blots were shown in Supplementary Fig. 3. **E** The upregulation of *BIRC5* was associated with a worse prognosis for patients with LUAD. **F** Univariable and multivariable Cox regression analysis showed that *BIRC5* was an independent risk factor of LUAD. G. The inverse correlation of *PTPRT* and *BIRC5* in multiple cancers. Data were shown as the mean ± SD (*n* = 3). * *P* < 0.05, ** *P* < 0.01, *** *P* < 0.001 by unpaired Student’s t test

Due to the unique role of *BIRC5* in adenocarcinoma [27, 28] and its strongest negative correlation with *PTPRT* expression, we then focused on the influence of modulation of *PTPRT* expression on *BIRC5*. Notably, the protein levels of *BIRC5* were decreased upon *PTPRT* overexpression in LUAD cells (Fig. 4A, B, Supplementary Fig. 1– 2), while the expression of *BIRC5* was upregulated at both mRNA (Fig. 4C) and protein (Fig. 4D, Supplementary Fig. 3) levels upon *PTPRT* knockdown in multiple cell lines. In addition, high expression of *BIRC5* was significantly correlated with a worse prognosis in the TCGA-LUAD cohort (HR = 1.75, 95% CI 1.31 ~ 2.35, *P* < 0.0001; Fig. 4E). After multivariable Cox regression analysis, *BIRC5* remained an independent risk factor for patients with LUAD (HR = 1.73, 95% CI 1.27 ~ 2.36, *P* = 0.0005, Fig. 4F). Furthermore, *PTPRT* was negatively correlated with *BIRC5* in other cancer types such as breast carcinoma (BRCA, R = -0.497, *P* < 0.0001; Fig. 4G), pancreatic adenocarcinoma (PAAD, R = -0.371, *P* < 0.0001; Fig. 4G), stomach adenocarcinoma (STAD, R = -0.279, *P* < 0.0001; Fig. 4G), and mesothelioma (MESO, R = -0.465, *P* < 0.001; Fig. 4G). Collectively, our *in-vitro* results underscored the essential roles of *PTPRT* in inhibiting cell proliferation by affecting the expression of cell cycle-related genes like *BIRC5*, indicating that *PTPRT* downregulation could promote the malignant proliferation of tumour cells.

### *PTPRT* suppressed cell migration and invasion

**Fig. 5:**
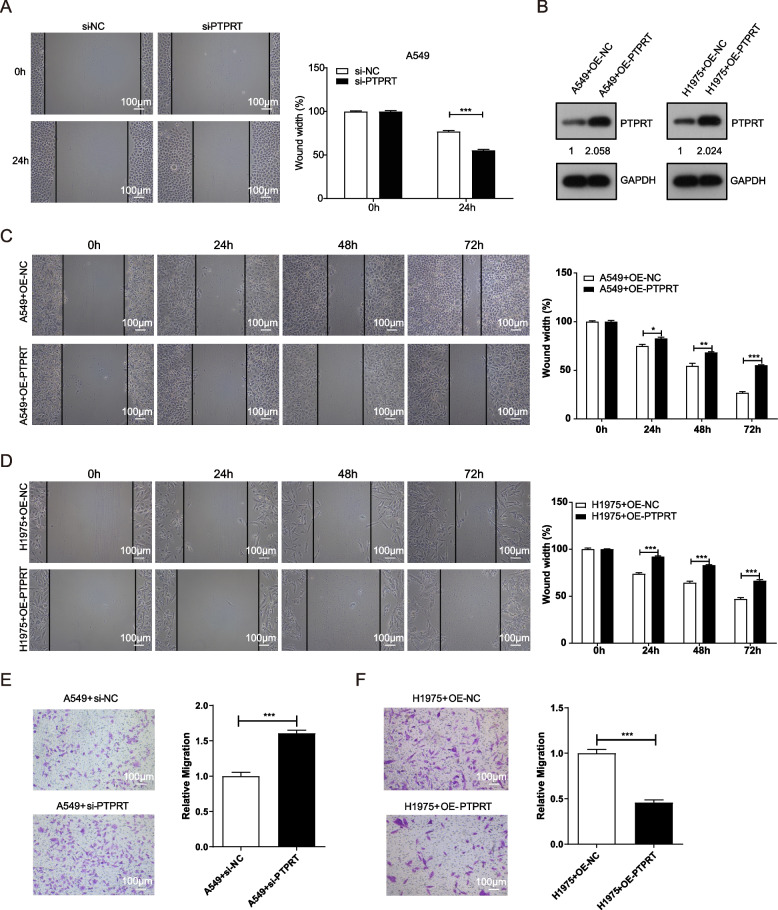
Wound healing assays of cell migration in A549 and H1975 cells. **A** Wound healing assays of si-NC and si-PTPRT in A549 cells. **B** Western blot images of PTPRT overexpression in A549 and H1975 cells, full-length gels and blots were shown in Supplementary Fig. 4. **C** Wound healing assays of Vector (OC-NC) and OE-PTPRT in A549 cells. **D** Wound healing assays of Vector (OC-NC) and OE-PTPRT in H1975 cells. Wound healing was recorded and quantitated for three microscopic fields. **E** Invasion of si-NC and si-PTPRT A549 cells were stained and counted under a light microscope. **F** Invasion of Vector (OE-NC) and OE-PTPRT H1975 cells were stained and counted under a light microscope. Scale bar, 100 µm. Data were shown as the mean ± SD (*n* = 3). * *P* < 0.05, ** *P* < 0.01, *** *P* < 0.001 by unpaired Student’s t test

We previously showed that the mutations in *PTPRT* were associated with the cancer metastasis of multiple cancer types [7]. To investigate whether downregulation of *PTPRT* was associated with cell migration and invasion, we performed wound healing and transwell assays using *PTPRT*-knocked-down or overexpressed tumour cells. Wound healing assays showed that *PTPRT* knockdown (si-PTPRT) promoted the migration capabilities of A549 cells (Fig. 5A; *P* = 8.059E-05). On the other hand, overexpression of *PTPRT* (OE-PTPRT) in A549 and H1975 cells significantly attenuated their migration capabilities (Fig. 5B-D). Consistently, transwell assays showed that *PTPRT* knockdown enhanced the invasion capabilities of A549 cells (Fig. 5E; *P* = 0.0009), and *PTPRT* overexpression significantly reduced the invasion capabilities of H1975 cells (Fig. 5F; *P* = 0.0005).

### *PTPRT* expression was associated with the prognosis of LUAD and other cancer types

**Fig. 6:**
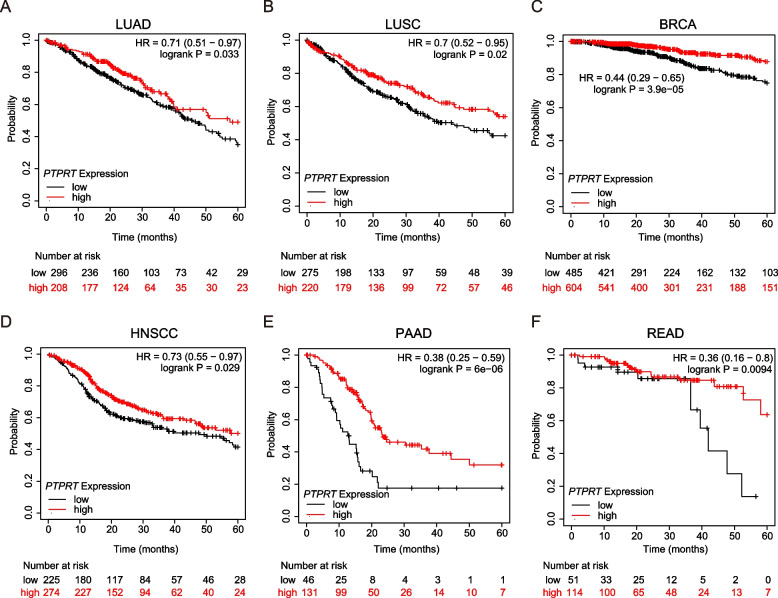
Kaplan–Meier survival curve of human cancers with high and low *PTPRT* expression. **A** LUAD, lung adenocarcinoma. **B** LUSC, squamous cell carcinoma of lung. **C** BRCA, breast carcinoma. **D** HNSCC, head and neck squamous cell carcinoma. **E** PAAD, pancreatic adenocarcinoma. **F** READ, Rectal adenocarcinoma. The survival analysis between PTPRT-high and PTPRT-low subgroups was performed on the “mRNA RNA-seq: Pancancer” module of the KM-plotter (http://kmplot.com)

As *PTPRT* showed regulatory effects on the proliferation, migration, and invasion of LUAD cells, we speculated that the expression level of *PTPRT* might be predictive for the prognosis of patients with LUAD. Using KM-plotter [20], a web-based survival analysis tool tailored for medical research, we found that high expression of *PTPRT* was significantly correlated with better survival rates of patients with LUAD (HR = 0.71, %95 CI 0.51 ~ 0.97, log-rank *P* = 0.033; Fig. 6A). Similarly, high expression of *PTPRT* was also significantly correlated with favourable prognosis of patients with LUSC (HR = 0.7, 95% CI 0.52 ~ 0.95, *P* = 0.02; Fig. 6B), breast carcinoma (HR = 0.44, 95% CI 0.29 ~ 0.65, *P* = 3.9E-5; Fig. 6C), head and neck squamous cell carcinoma (HNSCC, HR = 0.73, 95% CI 0.55 ~ 0.97, *P* = 0.029; Fig. 6D), pancreatic adenocarcinoma (PAAD, H = 0.38, 95% CI 0.25 ~ 0.59, *P* = 6E-6; Fig. 6E), and rectum adenocarcinoma (READ, HR = 0.36, 95% CI 0.16 ~ 0.8, *P* = 0.0094; Fig. 6F).

### *PTPRT* expression was associated with elevated TMB and TNB in lung cancer

**Fig. 7:**
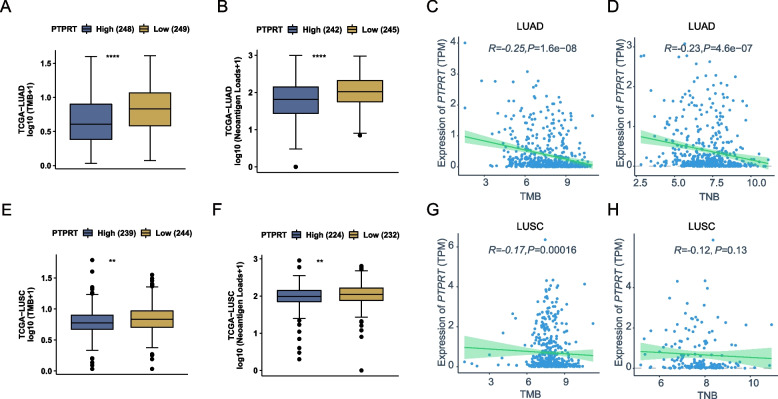
The association of *PTPRT* expression with TMB and TNB. TMB (**A**) and TNB (**B**) difference between PTPRT-high and PTPRT-low samples in TCGA-LUAD. TMB (**C**) and TNB (**D**) were negatively correlated with *PTPRT* expression level in TCGA-LUAD. TMB (**E**) and TNB (**F**) difference between PTPRT-high and PTPRT-low samples in TCGA-LUSC. TMB (**G**) and TNB (**H**) were negatively correlated with *PTPRT* expression levels in TCGA-LUSC. Spearman correlation was determined

Previous studies found that the occurrence of *PTPRT* mutation was associated with a high TMB and better responses to ICI therapies [16]. To explore whether the expression level of *PTPRT* was also associated with TMB, we divided the TCGA-LUAD cohort into the PTPRT-high subgroup and PTPRT-low subgroup using the median expression of *PTPRT* as a cut-off, and found that the PTPRT-high subgroup had a lower TMB and TNB than the PTPRT-low subgroup (Fig. 7A, B). The *PTPRT* expression level was also negatively correlated with TMB and TNB, respectively (Fig. 7C, D, Table S7- 8). Similar analysis was performed with the TCGA-LUSC cohort, and the results were in accordance with those in LUAD (Fig. 7E–H, Table S9- 10). Together, these findings suggested that the expression level of *PTPRT* was associated with tumour immunogenicity in lung cancer, and might be predictive of the responses to ICI therapies.

## Discussion and conclusion

Previous studies have shown that *PTPRT* functioned as a tumour suppressor involved in cell–cell aggregation and tumour progression [29–31]. Peyser et al. found that hypermethylation in the promoter region of *PTPRT* as well as mutations in *PTPRT* promoted the activation of the STAT3 pathway [1, 32]. In our previous studies, we found that *PTPRT* mutations were associated with cancer metastasis and *PTPRT* expression was downregulated in a variety of cancers, including lung cancer [7, 33]. However, the specific effects of *PTPRT* downregulation remained largely unknown.

In this study, through analysis of TCGA gene expression data, we revealed that genes associated with the downregulation of *PTPRT* in LUAD were enriched in cell cycle pathways. In vitro functional verification using LUAD cell lines further demonstrated that modulation of *PTPRT* expression affected cell proliferation profoundly through the regulation of cell cycle-related genes. Notably, we showed that *BIRC5*, an oncogene specially involved in adenocarcinoma [27, 28], was significantly negatively correlated with *PTPRT* in LUAD, but not in LUSC. *BIRC5* acted as an important regulator of cell division, through directing the movement of passage protein complex (CPC) to different locations from the inner centromere [34, 35]. The expression of *BIRC5* was high during fetal development and in adenocarcinoma tissues, yet low in adult tissues [23, 36]. Our results showed that knockdown of *PTPRT* elevated the expression of *BIRC5* and promoted the proliferation, migration, and invasion of LUAD cells. On the other hand, overexpression of *PTPRT* suppressed *BIRC5* expression as well as the proliferation, migration, and invasion of LUAD cells, suggesting that *PTPRT* downregulation in tumours could promote the malignant growth or metastasis of lung cancer.

In recent years, ICI therapy have emerged as potent treatment strategies for various solid cancers [37, 38]. Many studies have been devoted to exploring biomarkers for predicting therapeutic responses to ICIs, among which TMB, PD-L1, and microsatellite instability (MSI) have been proven to be useful in practice [13, 39, 40]. Previous studies have shown that *PTPRT* mutation was positively correlated with the level of TMB, and patients with *PTPRT* mutation had higher response rates to ICIs than those with wild-type *PTPRT* [16, 17]. In this study, we found that *PTPRT* downregulation correlated with higher TMB and TNB in lung cancer, suggesting its role as a candidate biomarker for ICI therapy efficacies.

In conclusion, our findings uncovered the central role of *PTPRT* in regulating the expression of cell cycle-related genes including *BIRC5*. *PTPRT* downregulation promoted the proliferation, migration, and invasion of LUAD cells, while *PTPRT* overexpression exerted opposite effects. Additionally, we emphasized the predictive value of *PTPRT* expression on the clinical outcomes as well as tumour immunogenicity of lung cancer. Our work suggested that *PTPRT* expression might stratify patients with lung cancer for the use of ICIs, and new treatment tools might be developed through increasing *PTPRT* expression in LUAD.

### Supplementary Information

**Additional file 1:** **Supplementary Table S1.** Gene primers used in this study. **Supplementary Table S2.** siRNA sequence used in this study. **Supplementary Table S3.** Different expressed genes (DEGs) between PTPRT-high and PTPRT-low subgroups in TCGA-LUAD cohort. **Supplementary Table S4.** DEGs between PTPRT-high and PTPRT-low subgroups in TCGA-LUSC cohort. **Supplementary Table S5.** KEGG enrichment of DEGs (*N*=1376) specific to LUAD cohort. **Supplementary Table S6.** Gene Ontology enrichment of DEGs (*N*=1376) specific to LUAD cohort. **Supplementary Table S7.** The TMB and TPM of each sample in TCGA LUAD cohort. **Supplementary Table S8.** The TNB and TPM of each sample in TCGA LUAD cohort. **Supplementary Table S9.** The TMB and TPM of each sample in TCGA LUSC cohort. **Supplementary Table S10.** The TNB and TPM of each sample in TCGA LUSC cohort.

**Additional file 2:** **Supplementary Figure 1.** Overexpression of *PTPRT* in A549 cells led to decreased expression of BIRC5 (survivin) at the protein level. Three replicates of Western blots were performed. Notably, the marker (Shanghai Yase Biomedical Technology Co., LTD., WJ102) was not able to be developed. Therefore, the position of the marker on the Kodak film after exposure was determined by comparing the color bands on the acetate fiber film using SDS-PAGE horizontal electrophoresis. **Supplementary Figure 2.** Overexpression of *PTPRT* in H1975 cells led to decreased expression of BIRC5 at the protein level. Three replicates of Western blots were performed. **Supplementary Figure 3.** Knockdown of *PTPRT* led to decreased expression of BIRC5 at the protein level in A549, H838, and BEAS-2B cells. Three replicates of Western blots were performed in A549 cells and once each in H1299 and BEAS-2B. **Supplementary Figure 4.** Western blot image of PTPRT overexpression in A549 and H1975 cells. The experiment was repeated once in each cell line.

## Data Availability

The data reported in this study can be found in supplementary materials, and additional data can be obtained by contacting corresponding authors. The code used in this manuscript is available at: https://github.com/gkdsuperchan/PTPRT-expression.
