# Supplementary material for: Downregulation of PTPRT elevates the expression of survivin and promotes the proliferation, migration, and invasion of lung adenocarcinoma
Source: BMC Cancer. 2024 Jan 12;24:63. doi: 10.1186/s12885-024-11840-7 (PMC10785488; doi:10.1186/s12885-024-11840-7)
Supplement: Supplementary file 2 — Additional file 2: Supplementary Figure 1. Overexpression of PTPRT in A549 cells led to decreased expression of BIRC5 (survivin) at the protein level. Three replicates of Western blots were performed. Notably, the marker (Shanghai Yase Biomedical Technology Co., LTD., WJ102) was not able to be developed. Therefore, the position of the marker on the Kodak film after exposure was determined by comparing the color bands on the acetate fiber film using SDS-PAGE horizontal electrophoresis. Supplementary Figure 2. Overexpression of PTPRT in H1975 cells led to decreased expression of BIRC5 at the protein level. Three replicates of Western blots were performed. Supplementary Figure 3. Knockdown of PTPRT led to decreased expression of BIRC5 at the protein level in A549, H838, and BEAS-2B cells. Three replicates of Western blots were performed in A549 cells and once each in H1299 and BEAS-2B. Supplementary Figure 4. Western blot image of PTPRT overexpression in A549 and H1975 cells. The experiment was repeated once in each cell line. [file 12885_2024_11840_MOESM2_ESM.docx]

**Supplementary Figures**

**Supplementary Figure 1.** Related to Figure 4A.


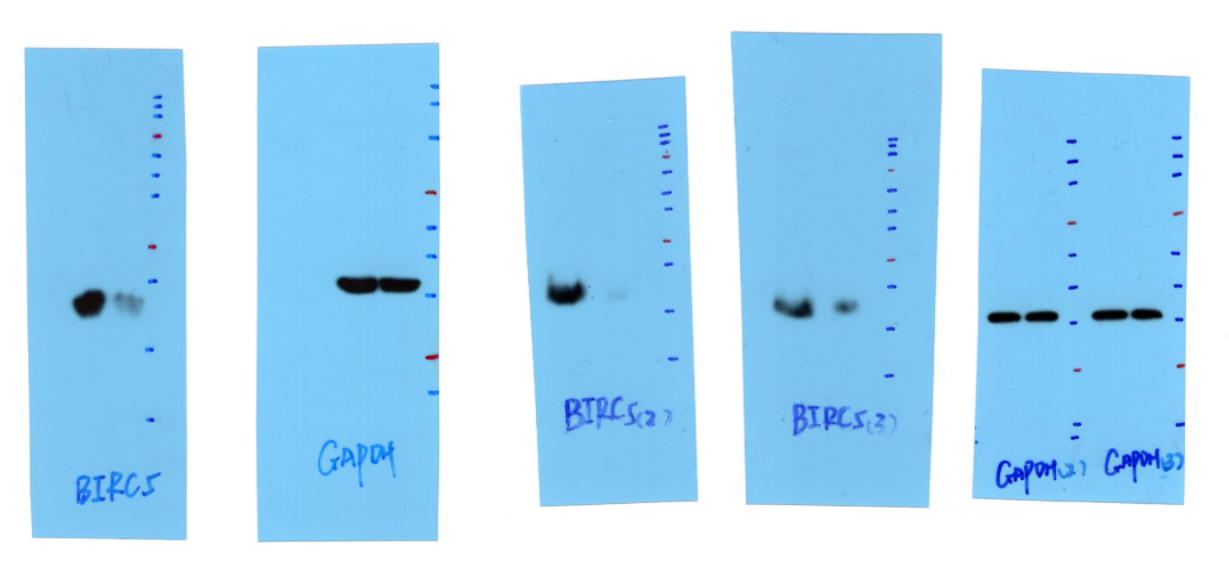


Supplementary Figure 1. Overexpression of *PTPRT* in A549 cells led to decreased expression of BIRC5 (survivin) at the protein level. Three replicates of Western blots were performed. Notably, the marker (Shanghai Yase Biomedical Technology Co., LTD., WJ102) was not able to be developed. Therefore, the position of the marker on the Kodak film after exposure was determined by comparing the color bands on the acetate fiber film using SDS-PAGE horizontal electrophoresis.

**Supplementary Figure 2.** Related to Figure 4B.


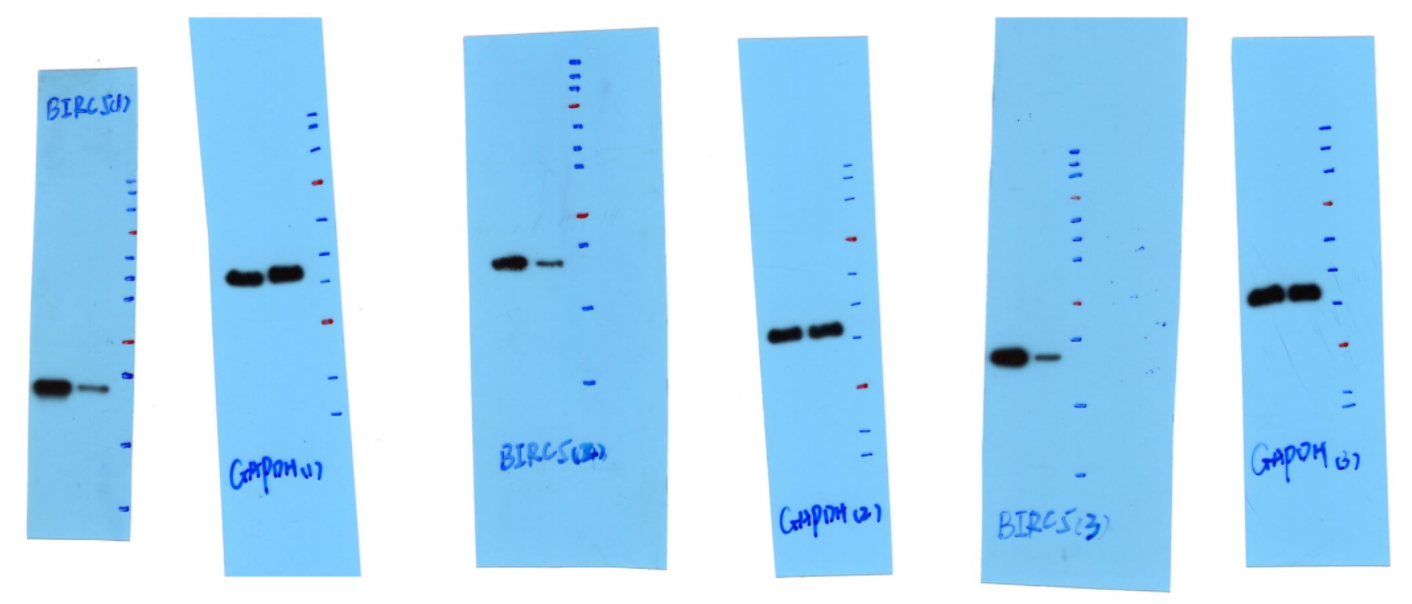


Supplementary Figure 2. Overexpression of *PTPRT* in H1975 cells led to decreased expression of BIRC5 at the protein level. Three replicates of Western blots were performed.

**Supplementary Figure 3.** Related to Figure 4D.


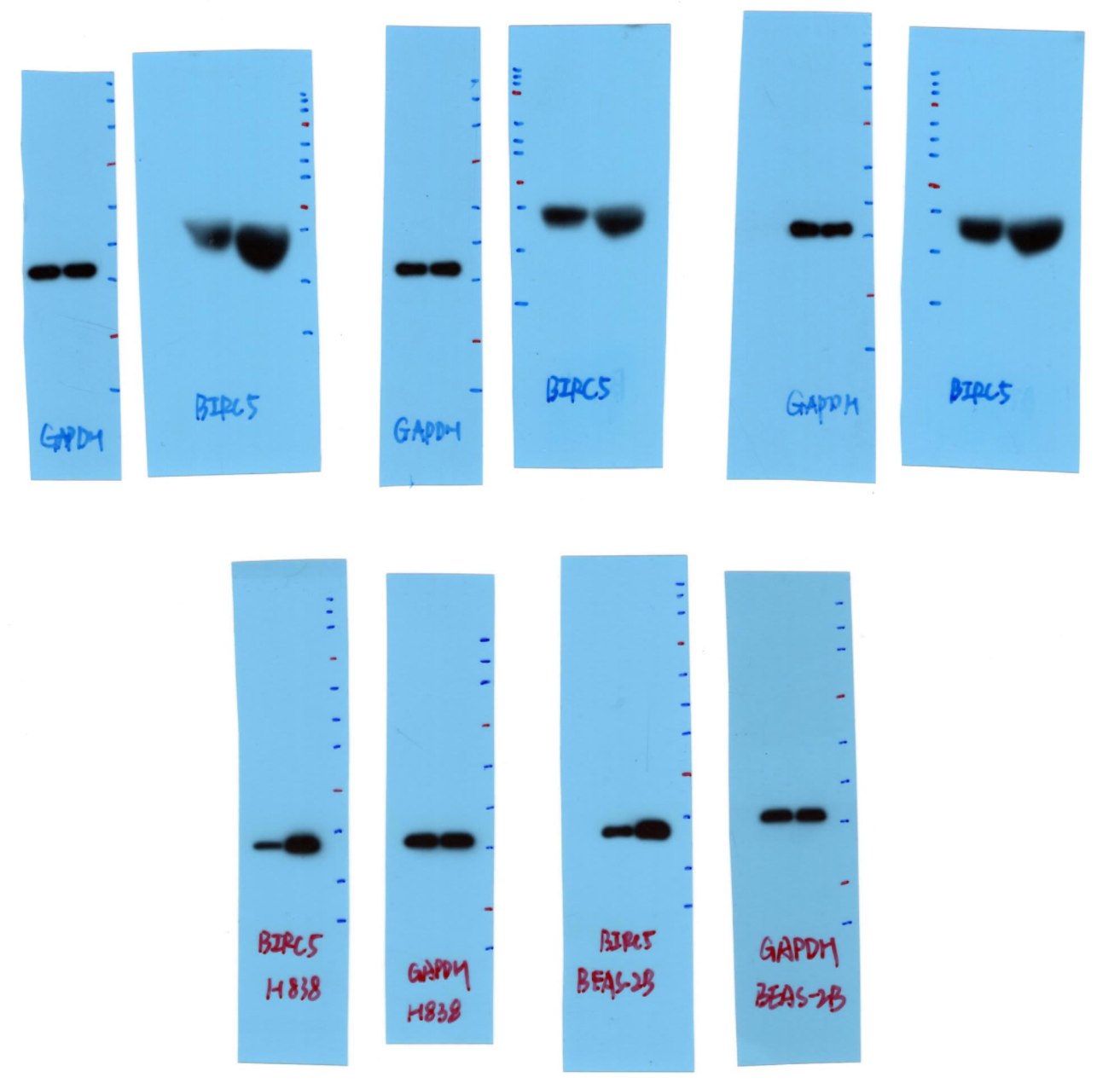


Supplementary Figure 3. Knockdown of *PTPRT* led to decreased secretion of BIRC5 at the protein level in A549, H838, and BEAS-2B cells. Three replicates of Western blots were performed in A549 cells and once each in H1299 and BEAS-2B.

**Supplementary Figure 4.** Related to Figure 5B.


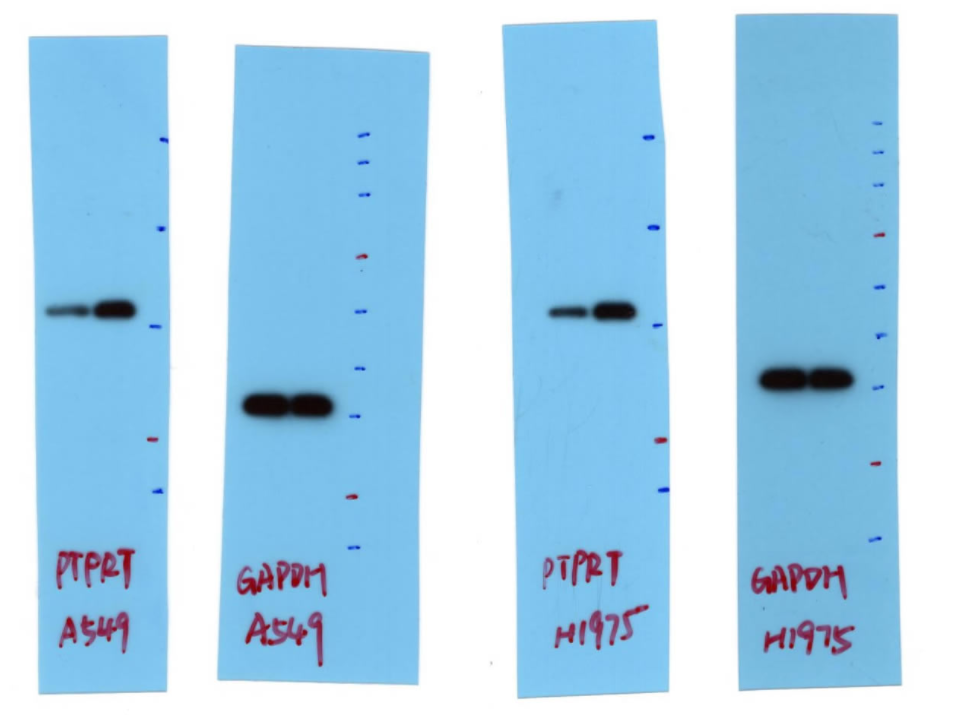


Supplementary Figure4. Western blot image of PTPRT overexpression in A549 and H1975 cells. The experiment was repeated once in each cell line.
